# Longitudinal changes of lactopontin (milk osteopontin) in term and preterm human milk

**DOI:** 10.3389/fnut.2022.962802

**Published:** 2022-07-29

**Authors:** Jing Zhu, Xue Yu, Yiran Wang, Shasha Bai, Jianqiang Lai, Xiaomei Tong, Yan Xing

**Affiliations:** ^1^Beijing Institute of Nutritional Resources, Beijing Academy of Science and Technology, Beijing, China; ^2^School of Public Health, Peking University Health Science Center, Beijing, China; ^3^National Institute for Nutrition and Health, Chinese Center for Disease Control and Prevention, Beijing, China; ^4^Department of Pediatrics, Peking University Third Hospital, Beijing, China

**Keywords:** osteopontin, lactopontin, breast milk, infant, preterm

## Abstract

**Background:**

Lactopontin (LPN) in breast milk, also known as milk osteopontin is thought to play a myriad of important roles in infants when they are immature. The purpose of the present study was to examine the longitudinal changes in LPN concentrations in term and preterm milk, and elucidate the links between maternal characteristics, LPN levels, and child growth in a birth cohort.

**Methods:**

131 mothers who delivered term, moderate-late preterm (MPT), very preterm (VPT), and extremely preterm (EPT) infants were included, milk samples were collected at 7, 14, 28, and 120 days postpartum. LPN concentration was determined by multiple reaction monitoring (MRM) using ultra-performance liquid chromatography-tandem mass spectrometry (UPLC-MS/MS).

**Results:**

Our results indicated that LPN change over time of VPT (*P* = 0.024) and EPT (*P* = 0.003) were significantly different from term milk, although they all gradually decreased with lactation. In terms of LPN-related factors, maternal age was a significant contributor in late mature milk and pre-pregnancy BMI a significant contributor to colostrum and transitional milk. We further investigated relationships between LPN levels and infant weight and our results suggested that high levels of LPN in breast milk might be useful for the catch-up growth of infants.

**Conclusion:**

LPN levels in breast milk are related to maternal factors, and differences in LPN levels may affect the growth of infants. As milk is a critical part in the mother–breastmilk–infant “triad,” the association between maternal-infant factors and milk LPN levels warrants further study.

## Introduction

Human milk provides multiple molecular components to guarantee the best start for an infant at a healthy life (1). Among the many different components (lactose, human milk oligosaccharides, lipids, etc.), proteins are crucial nutritional and bioactive molecular factors in human milk. In recent years, osteopontin (OPN) in milk, also known as lactopontin (LPN) (2, 3), has attracted much attention. OPN was first identified in bone tissue as a bone matrix protein (4, 5). OPN was further discovered in various tissues and cells in two forms: intracellular OPN found in immune cells (6) and secreted OPN present in body fluid (7). LPN is a unique type of OPN as its concentration in human milk, especially in colostrum, was much higher than other types of OPN in other tissues and excretions (8). Moreover, LPN showed a higher degree of posttranslational modifications (PTMs) than other types of OPN (9–11).

The high level of LPN in colostrum indicates that LPN may play some roles when the infants are immature (12). Recent studies have discovered a myriad of biological roles of LPN for the development and health of infants. LPN is capable to stabilize calcium by forming a soluble complex with calcium (13). Due to its integrins and receptors binding properties, LPN shows bioactivities in intestinal development, brain development, and immunological development (14).

Preterm birth is defined with gestational age less than 37 weeks. Globally, approximately 15 million preterm infants are born per year and preterm birth complications are the leading contributors to the deaths of children under 5 years of age (15, 16). Compared to term infants, preterm infants have higher vulnerable body surfaces, and more immature organ systems due to shorter intrauterine time (17). Preterm birth can be subdivided based on gestational age: extremely preterm (EPT, less than 28 weeks); very preterm (VPT, 28–32 weeks) and moderate-late preterm (MPT, 32–37 weeks). The shorter the gestational age is, the worse the developmental status of preterm infants (18). Preterm infants, especially the extremely preterm and very preterm infants, are usually accompanied by immature gut and immune system, and therefore, are prone to short- and long-term health consequences, such as infections, growth concerns, developmental impairments, and other comorbidities (19). As suggested in animal studies, LPN might play a critical role in preterm birth. Supplementary LPN was demonstrated to reduce the rate of diarrhea in preterm pigs (20) and, while it did not affect the incidence of necrotizing enterocolitis, it can reduce the severity (21). LPN was identified in preterm breast milk by bottom-up mass spectrometry-based approach (22, 23). However, to the authors’ knowledge, LPN concentration in preterm milk, especially the longitudinal changes among the various preterm subcategories was not reported yet.

The purpose of the present study was to examine the longitudinal changes in LPN concentrations in term, MPT, VPT, and EPT milk, and elucidate the links between maternal characteristics, LPN levels, and child growth in a birth cohort. Milk samples were collected at four-time points covering the lactational stages of colostrum (<7 days postpartum), transitional milk (8–14 days postpartum), early mature milk (15–28 days postpartum) and fully mature milk (> 42 days postpartum). LPN concentration was determined by multiple reaction monitoring (MRM) using ultra-performance liquid chromatography-tandem mass spectrometry (UPLC-MS/MS).

## Materials and methods

### Study population

**FIGURE 1:**
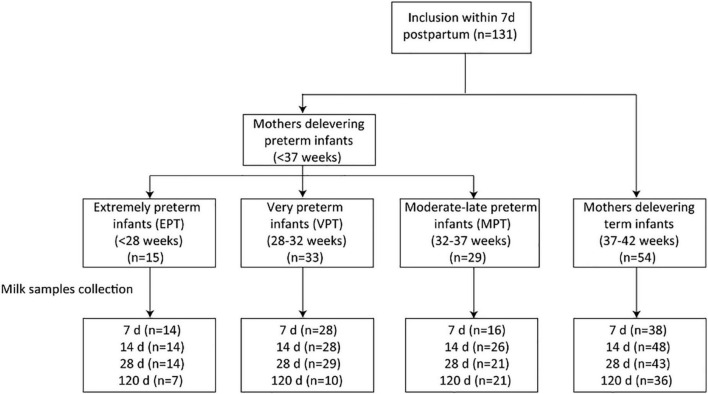
Flow chart of the study cohort.

This study was conducted between December 2017 and April 2020. Women who gave birth to healthy term or preterm infants were recruited from the Department of Pediatrics, Peking University Third Hospital. Eligible criteria included women aged 18 years and older, and with the intention to breastfeed for at least 4 months postpartum. Women who delivered infants with congenital malformations, genetic diseases or required surgery were excluded. A total of 131 women were included. Participants were further grouped into four, according to their infants’ gestational age at birth: extremely preterm (EPT, less than 28 weeks); very preterm (VPT, 28–32 weeks); moderate-late preterm (MPT, 32–37 weeks) and full-term (37–42 weeks) (24). The study flow chart is depicted in Figure 1.

Baseline information of both mothers and infants were collected through the electronic medical record system, which included maternal height, weight, age, parity, mode of delivery, and infants gestational age and birthweight. Infant weight was obtained from questionnaires completed by parents at 28 and 120 days postpartum, which was accurate to 0.1 kg. Fenton preterm growth charts (25) were used to calculates Z-scores for weight of preterm infants to 49 weeks and WHO child growth standard for term infants and preterm infants after 50 weeks.

This study was approved by the Peking University Third Hospital Medical Science Research Ethics Committee (ethical approval reference: S2016159). All mothers who participated in this study signed the written informed consent.

### Milk collection

Breast milk samples were collected from the mothers at 7, 14, 28, and 120 days after delivery. A trained nurse performed the milk expression during hospitalization. Mothers were trained to collect breast milk at home when discharged. The breast milk samples were collected between 9:00 and 11:00 in the morning before breastfeeding. Mothers are told to wash their hands and clean their breasts before collecting breast milk. After that, they need to manually express or pump full milk expression from a single breast and then homogenized. An aliquot of 10 mL was taken for analysis in collection tubes, and the rest was used to feed the infants. The collected breast milk was temporarily stored in a refrigerator at home and transferred to the hospital as soon as possible within 1–2 days with ice packs, and stored at –80^°^C until thawed for analysis.

### Targeted multiple reaction monitoring analysis for lactopontin

#### Selection of lactopontin signature peptide

For the targeted MRM analysis, the potential signature peptides were selected by peptides identified in our previous study of bottom-up milk proteome (26). The general rules for targeted proteomics were applied for further selection. In brief, we attempted to avoid peptides which were too short (less than six amino acids) or too long (more than 25 amino acids), contained Met residues or sites for potential modification, and peptides with miscleavege (27). Additionally, the specificity of potential signature peptides was proved by an online BLAST search in NCBI.^1^ After exploration, the signature peptide to detect LPN was determined and the sequence was GDSVVYGLR.

#### Chemicals and reagents

The signature peptide GDSVVYGLR (purity was 99.01%) and the synthetic isotopic peptide GDSVVYGLR*(purity was 99.63%) were synthesized by GLS Co., Ltd. (Shanghai, China). Analytical grade sodium bicarbonate (NaHCO3) and calcium chloride (CaCl2) were purchased from XILONG SCIENTIFIC Co., Ltd. (Guangzhou, China). Analytical grade acetic acid (HAC) was purchased from BEIJING HUAGONG Co., Ltd. (Beijing, China). HPLC grade dithiothreitol (DTT), iodoacetamide (IAA) were purchased from Sigma-Aldrich (St. Louis, MO, United States). LC-MS grade formic acid (FA) and LC/MS grade acetonitrile (ACN) were purchased from Fisher Chemical (MA, United States). Ultrapure water was generated from a water purification system (Aquaplore, United States). Mass spectrometry grade trypsin was purchased from Promega USA.

#### Sample preparation

The breast milk samples were thawed at 4^°^C. Before tryptic digestion, 20 μL milk sample was mixed with 180 μL of 500 mM NaHCO_3_ and 20 μL 500 mM DTT and the mixture was incubated in a 70^°^C water bath for 30 min. Alkylation was then performed by adding 60 μL of 500 mM IAA for 30 min at room temperature in the dark. Subsequently, 10 μL of 100 mM CaCl_2_ and 100 μL of 200 μg/mL trypsin were added and incubated in 37^°^C water bath for 4 h. The reaction was terminated by the addition of 10 μL of 100% formic acid. After 20 min, 40 μL of 1 μg/mL synthetic isotopic peptide solution and 570 μL of ultrapure water was added to reach a final volume of 1 mL. The mixture was cleaned by a 0.22 μm nylon filter for LC-MS/MS analysis.

#### Ultra-performance liquid chromatography-tandem mass spectrometry analysis

The UPLC-MS/MS experiments were performed on an ACQUITY UPLC system coupled to TQ-S with electrospray ionization (ESI) (Waters, Milford, MA, United States). The analytical column was Waters XBridge Peptide BEH C18 column (1.7 μm, 2.1 × 100 mm, 300 Å) and the temperature was kept at 35^°^C. The mobile phase consisted of 0.1% FA aqueous solution (Solvent A) and ACN (Solvent B). The flow rate was 300 μL/min. The gradient consisted of an initial 20%B for 1 min, followed by an increase from 20 to 40%B over 4 min, and followed by a second increase from 40 to 100%B over 0.5 min, the gradient was then decreased to 20%B over 0.5 min and maintained at 20%B for 1 min. The total chromatography time was 7 min and the injection volume was 5 μL.

The quantitative analysis was performed in MRM mode in duplicates. Mass transitions monitored in the analysis were m/z 483.2 > 345.1 and 483.2 > 607.3 for the signature peptide GDSVVYGLR; and m/z 483.2 > 355.1 and 483.2 > 617.3 for the isotope internal standard peptide GDSVVYGLR*. The synthetic peptide GDSVVYGLR was used to generate the standard curve of intensity vs. concentration. The mass spectrometer parameters were as follows: capillary voltage at 3.0 kV, cone voltage at 40 V, the dissolvent temperature at 350^°^C, dissolvent gas flow at 700 L/min, tapered hole back blowing gas flow at 150 L/h, collision chamber pressure at 7 bar. More than fifteen points were collected for each peak.

### Statistical analysis

Statistical analyses were performed using R statistical software version 4.0.3. Baseline characteristics of the mothers and the infants were presented as mean ± *SD* for continuous variables and frequencies for categorical variables. Continuous variables were analyzed between term and preterm using 2-sample *t*-tests, and between four groups (term, moderate-late preterm, very preterm, and extremely preterm milk) using ANOVA tests. Categorical data were analyzed using Pearson χ^2^ tests.

To compare the LPN level between three preterm groups and term infants, the term group was taken as reference, and three preterm groups were compared with it separately. To take advantage of the longitudinal design and repeated measurement of LPN, generalized estimating equation (GEE) was used to estimate the main effect and interaction between LPN level and time. If the interaction is significant, the simple effects at each time point were tested using Wilcoxon rank-sum test since the data was not normally distributed, indicated by the Shapiro-Wilk test.

To validate associations between LPN concentrations and maternal characteristics, Pearson correlation was used for continuous variables and Spearman correlation for categorical variables. Pearson correlation was used to validate associations between LPN concentrations and infants’ growth, including weight z-scores and weight gain z-scores.

In all statistical tests, results were interpreted as statistically significant where p was less than 0.05.

## Results

### Study population

The general characteristics of the study population are presented in Table 1. In total 131 participants were enrolled in the study. As we divided the participants into 4 groups (term, MPT, VPT, and EPT) based on their gestational ages, it was expected that the gestational ages among these groups were significantly different. The average age of the mother participants was 33.58 ± 4.29 years old, and no significant difference in maternal age was observed among the four groups. The average body mass index (BMI) before pregnancy was 22.50 ± 3.02 and showed no difference among the four groups. As expected, mothers in preterm groups tended to deliver by C-section and give birth to twins and even triplets. Other maternal factors, such as maternal parity, education background, were similar among mothers who delivered term, and preterm babies.

**TABLE 1:** Baseline characteristics of the mothers and infants.

	Term	Preterm				P1	P2
		Total	MPT	VPT	EPT		
Mothers (*n*)	54	77	29	33	15		
Gestational age at delivery (week)	38.9 ± 1.1	30.8 ± 3.1	34.0 ± 1.0	30.1 ± 1.3	26.1 ± 0.7	<0.001**	<0.001**
Maternal age (year)	33.5 ± 4.2	33.5 ± 4.4	33.3 ± 3.8	33.4 ± 5.2	34.2 ± 3.7	0.926	0.934
Maternal pre-pregnancy BMI (kg/m^2^)	22.3 ± 3.2	22.9 ± 3.1	22.8 ± 2.8	22.8 ± 3.3	23.2 ± 3.4	0.293	0.747
Mode of delivery (*n*, %)							
Vaginal	28 (51.9%)	27 (35.1%)	6 (20.7%)	9 (27.3%)	12 (80.0%)	0.055	<0.001**
C-section	26 (48.1%)	50 (64.9%)	23 (79.3%)	24 (72.7%)	3 (20.0%)		
Multiple births (*n*, %)							
Singleton	53 (98.1%)	45 (58.4%)	14 (48.3%)	23 (69.7%)	8 (53.3%)	<0.001**	<0.001**
Twins and triplets	1 (1.9%)	32 (41.6%)	15 (51.7%)	10 (30.3%)	7 (46.7%)		
Maternal parity (*n*, %)							
1	33 (61.1%)	51 (66.2%)	19 (65.5%)	20 (60.6%)	12 (80.0%)	0.547	0.579
> 1	21 (38.9%)	26 (33.8%)	10 (34.5%)	13 (39.4%)	3 (20.0%)		
Mother’s education (*n*, %)							
High school or less	0 (0.0%)	11 (14.3%)	3 (10.3%)	6 (18.2%)	2 (13.3%)	0.001**	0.053
Undergraduate	30 (55.6%)	46 (59.7%)	18 (62.1%)	19 (57.6%)	9 (60.0%)		
Postgraduate or above	24 (44.4%)	20 (26.0%)	8 (27.6%)	8 (24.2%)	4 (26.7%)		
Infants (*n*)	55	108	42	44	22		
Sex, boys (*n*, %)	38 (69.1%)	56 (51.9%)	23 (54.8%)	21 (47.7%)	12 (54.5%)	0.035*	0.175
Birthweight (g)	3301.6 ± 453.0	1530.3 ± 540.7	2002.9 ± 416.8	1344.3 ± 339.1	921.6 ± 109.4	<0.001**	<0.001**
Birth length (cm)	49.4 ± 2.0	39.6 ± 4.5	43.2 ± 3.2	38.3 ± 3.6	34.8 ± 2.1	<0.001**	<0.001**
Head circumference (cm)	34.3 ± 1.7	28.5 ± 3.2	31.0 ± 1.8	27.9 ± 2.5	24.3 ± 1.2	<0.001**	<0.001**
Apgar score at 1 min	9.9 ± 0.5	8.9 ± 1.6	9.6 ± 0.8	9.0 ± 1.5	7.4 ± 1.8	<0.001**	<0.001**
Apgar score at 5 min	10.0 ± 0.1	9.5 ± 0.7	9.9 ± 0.3	9.6 ± 0.7	8.7 ± 0.6	<0.001**	<0.001**

P_1_, Differences between Term and total preterm conducted by 2-sample t-test; P_2_, Differences between the four groups (term, moderate-late preterm, very preterm, and extremely preterm) conducted by ANOVA. Statistical differences were indicated by *P < 0.05, **P < 0.01.

For the infants, the birthweight of term infants were higher than preterm ones, especially the EPT infants only had an average birthweight of 957.69 ± 107.87 g. Other measurements for infants, including birth length, head circumference and Apgar score at 1 and 5 min, were worse for preterm infants. There was no difference in sex distribution.

### The lactopontin concentrations change over time in term, moderate-late preterm, very preterm, and extremely preterm milk

**FIGURE 2:**
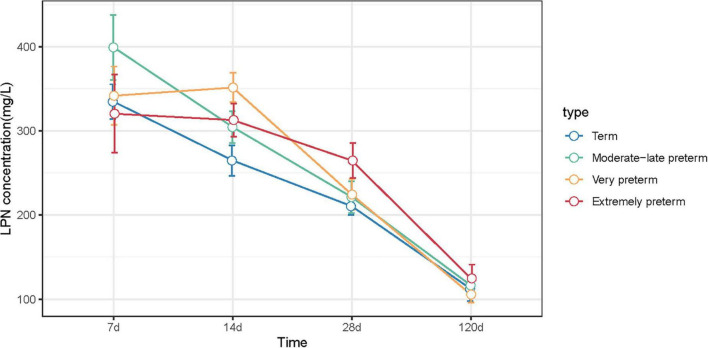
LPN concentrations of term, MPT, VPT, and EPT milk at 7, 14, 28, and 120 days postpartum. Statistical differences compared to term group were indicated by *P < 0.05, **P < 0.01.

As illustrated in Figure 2 and Supplementary materials, for both term and preterm milk (MPT, VPT, and EPT), LPN levels decreased dynamically throughout the lactational periods from colostrum to fully mature milk. The result of GEE indicated that LPN change over time of VPT (*P* = 0.024) and EPT (*P* = 0.003) were significantly different from term milk. We further compared the simple effects at each time point. At 14 days postpartum, the level of LPN in VPT milk (medium: 351.48 vs. 264.76 mg/L, *P* = 0.009) was significantly higher than term milk; at 28 days postpartum, LPN in VPT milk (224.33 vs. 210.45 mg/L, *P* = 0.038) and EPT milk (264.75 vs. 210.45 mg/L, *P* = 0.007) was significantly higher than term milk. However, no difference was observed between MPT and term milk. Our result indicated that mothers who delivered most immature infants tended to produce more LPN in early mature milk.

### Lactopontin levels in relation to maternal characteristics

The correlation between LPN levels and maternal characteristics was shown in Table 2. Maternal age was positively associated with LPN levels at 120 days postpartum. For the maternal pre-pregnancy BMI (kg/m2), it was positive associated with LPN levels at 7 and 14 days postpartum. No correlation was found between mode of delivery, multiple births, maternal parity, mother’s education background, and LPN levels.

**TABLE 2:** Maternal factors and their correlation with milk LPN levels.

Maternal factors	Lactation (days)	r	P_1_	P_2_
Maternal age (year)	7	0.183	0.083	–
	14	0.088	0.357	–
	28	0.121	0.223	–
	120	0.273	0.019*	–
Maternal pre-pregnancy BMI (kg/m^2^)	7	0.289	0.005**	–
	14	0.201	0.034*	–
	28	0.107	0.288	–
	120	0.216	0.073	–
Mode of delivery	7	0.003	–	0.977
	14	0.109	–	0.245
	28	0.103	–	0.290
	120	0.222	–	0.057
Multiple births	7	0.032	–	0.755
	14	0.108	–	0.247
	28	0.190	–	0.050
	120	0.113	–	0.337
Maternal parity	7	–0.007	–	0.945
	14	–0.123	–	0.189
	28	–0.130	–	0.183
	120	–0.034	–	0.775
Mother’s education	7	–0.082	–	0.430
	14	0.067	–	0.474
	28	0.005	–	0.959
	120	0.076	–	0.518

P_1_, Pearson correlation test; P_2_, Spearman correlation test. Statistical differences were indicated by *P < 0.05, **P < 0.01.

### Relationships between lactopontin levels and infant weight

In this study, we investigated the relationship between LPN concentrations and infant weight in all infants, as well as in term and preterm infants (Table 3). In total infants, LPN concentration was negatively associated with infant weight z-score at 28 days postpartum. However, this difference was not significant at 120 days postpartum, which suggested that high levels of LPN in breast milk might be useful for the catch-up growth of infants.

**TABLE 3:** Pearson correlation between LPN Levels and infant weight.

	Lactation (days)	r_infant weight z–*score*_	P_infant weight z–score_	r_infant weight gain z–score_	P_infant weight gain z–score_
Total	28	–0.243	0.041*	–0.071	0.558
	120	–0.060	0.610	0.103	0.380
Term	28	–0.040	0.815	0.298	0.074
	120	0.004	0.981	0.161	0.378
Preterm	28	–0.277	0.113	–0.213	0.234
	120	–0.201	0.197	0.005	0.976

Statistical differences were indicated by *P < 0.05.

## Discussion

This study measured LPN levels in term and preterm milk at four time points covering the four lactational stages of colostrum (< 7 days postpartum), transitional milk (8–14 days postpartum), early mature milk (15–28 days postpartum) and fully mature milk (> 42 days postpartum). To our knowledge, this study provides the first dataset of the longitudinal changes of LPN levels in preterm subcategories.

Currently, most published studies measured LPN levels by enzyme-linked immunosorbent assay (ELISA) (5, 28, 29). ELISA is widely used for biochemical analysis as its high specificity and sensitivity by utilizing the specific binding of antigen to antibody. However, data acquired from different commercially ELISA kits showed variable and non-comparable absolute concentrations (30). Moreover, the ELISA method is of concern since cross-reactivity or non-specific antibody recognition have been noticed in complex sample matrix and lead to false negative or false positive (31). Such problems have been observed in the article (28) mentioned that a study (5) detected LPN levels using an ELISA that was not validated for use in human milk, the detection levels of LPN was shown to overestimate up to 10-fold from the actual levels. Therefore, there is a need for more sensitive and accurate assays allowing for reliable detection of LPN in human milk. Recent studies, including our previous study, used a mass-spectrometry (MS)-based approach to confidently determine the presence of LPN protein (22, 26) or endogenous peptides (23, 32) in human milk, whereas these studies remain semiquantitative at best. Hereby, in this study, we quantified the LPN levels by targeted proteomics in MRM mode which allowed the consistent monitoring of peptides of interest with a high degree of specificity and sensitivity (27).

We found that the median levels of LPN in colostrum were higher than 300 mg/L, and decrease over time, which were similar for the term and the three preterm milk groups (i.e., MPT, VPT, and EPT). The LPN levels were discussed in several studies but the result contrasts. Jiang and Lönnerdal reported during the 1-year lactation period, the highest LPN content in colostrum was 178.0 mg/L, which dropped to 48.3 mg/L at 12 months of lactation (29). Bruun et al. found LPN levels varied across countries, from 99.7 mg/L in Danish, 185.0 mg/L in Japanese, 216.2 mg/L in Korean to 266.2 mg/L in Chinese mothers (28). The LPN levels in Chinese mothers’ breast milk in our study was in line with Bruun’s study.

However, the concentrations of LPN in term and preterm milk showed different trends over time. LPN levels were higher in transitional milk and early mature milk of VPT and EPT group, suggesting it may play important roles in preterm infants. Higher levels of LPN in colostrum and its decreasing trend indicates that LPN may be critical for early infancy (12). A randomized controlled trial of LPN supplementation suggested it may support improved innate and adaptive immune development, in which 240 healthy infants fed either standard formula or formula adding two doses of supplemental LPN or breast milk showed the benefit of LPN supplementation than standard formula in the plasma levels of several amino acids and cytokines, serum levels of the tumor necrosis factor-alpha (TNF-α), and LPN supplementation showed higher similarity to the breastfed infants in many aspects than infants fed standard formula alone (33, 34).

LPN levels might be affected by multiple factors like race and environment (diet, geographical factors, etc.) (28). Even in the same race, milk is known to be a dynamic biofluid reflecting the health status of maternal-infant dyads and forming the bridge between two interconnected individuals (35). Hereby, it is expected that we could notice a remarkable individual variance of milk LPN levels in our study. Our results have also demonstrated maternal age a significant contributor in late mature milk and pre-pregnancy BMI a significant contributor to colostrum and transitional milk. It was interesting that different maternal factors associated with LPN concentrations during specific lactation periods.

As milk is tailored to meet infants’ needs and a critical part in the mother–breastmilk–infant “triad” (36), the higher LPN level in these mothers may indicate the higher needs of LPN for their infants. We further investigated relationships between LPN levels and infant weight. Overall, LPN concentration was negatively associated with infant weight z-score at 28 days postpartum and this difference was not significant at 120 days postpartum. Our results suggested that high levels of LPN in breast milk might be useful for the catch-up growth of infants. However, Aksan et al. showed positive correlations between LPN levels and infants’ bodyweight and length at first and third months (37). However, in Aksan’s study only the LPN concentration in a single collection of breast milk was measured, so the relationship between LPN concentration and infant weight in the results was not derived from longitudinal data.

However, this work has some limitations to consider. First, since the study only investigated 131 women and their infants from one hospital, the results of this study needs to be confirmed in a broader study population. Second, the different sample sizes between 4 groups, and loss of participants during the 4-month follow-up period, limited the power of statistical analysis. Loss of follow-up was more common in the preterm groups than in the term group, especially in extremely preterm group and very preterm group. Therefore, when analyzing the relationship between LPN levels and the growth of infants, we combined the preterm groups. Third, the growth and development of breastfed infants are mainly affected by the total energy in breast milk, so the correlations of LPN levels with infant weight in this study cannot exclude the influence of confounding factors. Additional studies are required to clarify these relationships further.

## Conclusion

In summary, we quantified the LPN levels by targeted proteomics in MRM mode with a high degree of specificity and sensitivity. Our results indicated that the concentrations of LPN in term and preterm milk showed different trends over time, although both gradually decreased with lactation. In terms of LPN-related factors, maternal age was a significant contributor in late mature milk and pre-pregnancy BMI a significant contributor to colostrum and transitional milk. We further investigated relationships between LPN levels and infant weight and our results suggested that high levels of LPN in breast milk might be useful for the catch-up growth of infants. As milk is a critical part in the mother–breastmilk–infant “triad,” the association between maternal-infant factors and milk LPN levels warrants further study, especially in preterm infants.

## Data availability statement

The original contributions presented in this study are included in the article/Supplementary material, further inquiries can be directed to the corresponding author/s.

## Ethics statement

The studies involving human participants were reviewed and approved by the Peking University Third Hospital Medical Science Research Ethics Committee. Written informed consent to participate in this study was provided by the participants’ legal guardian/next of kin.

## Author contributions

XY and XT: conceptualization and investigation. JZ and SB: methodology. JZ, YX, and YW: formal analysis. JZ and YX: writing—original draft preparation. JL, XY, and XT: writing—review and editing. JZ: visualization. YX and XT: project administration. All authors have read and agreed to the published version of the manuscript.

## Footnotes

1
www.ncbi.nlm.nih.gov
