# Supplementary material for: Longitudinal changes of lactopontin (milk osteopontin) in term and preterm human milk
Source: Front Nutr. 2022 Jul 29;9:962802. doi: 10.3389/fnut.2022.962802 (PMC9372532; doi:10.3389/fnut.2022.962802)
Supplement: Supplementary file 1 [file Table_1.DOCX]

Supplementary table 1 LPN levels in 4 lactation stages of term, MPT, VPT and EPT milk

| Gestational age | Lactation | n | LPN level (mg/L) | | | | |
| --- | --- | --- | --- | --- | --- | --- | --- |
|  |  |  | medium | p25 | p75 | Min | Max |
| Term | 7d | 38 | 334.71 | 260.39 | 481.60 | 147.03 | 618.46 |
|  | 14d | 48 | 264.76 | 233.63 | 341.12 | 156.29 | 825.53 |
|  | 28d | 43 | 210.45 | 169.90 | 260.39 | 91.08 | 359.65 |
|  | 120d | 36 | 112.10 | 86.39 | 154.40 | 59.23 | 479.21 |
| MPT | 7d | 16 | 399.23 | 207.25 | 471.64 | 84.22 | 592.16 |
|  | 14d | 26 | 304.49 | 242.12 | 423.13 | 130.46 | 454.74 |
|  | 28d | 21 | 221.27 | 181.26 | 274.40 | 54.11 | 481.23 |
|  | 120d | 21 | 116.42 | 103.44 | 155.66 | 67.38 | 212.05 |
| VPT | 7d | 28 | 341.75 | 291.47 | 469.64 | 89.95 | 912.61 |
|  | 14d | 28 | 351.48 | 258.14 | 399.12 | 153.62 | 573.27 |
|  | 28d | 29 | 224.33 | 192.21 | 303.61 | 139.72 | 565.3 |
|  | 120d | 10 | 105.56 | 96.00 | 150.63 | 92.4 | 169.19 |
| EPT | 7d | 14 | 320.36 | 243.59 | 408.00 | 124.51 | 758.53 |
|  | 14d | 14 | 312.86 | 252.30 | 353.42 | 185.6 | 445.53 |
|  | 28d | 14 | 264.75 | 222.42 | 348.63 | 127.34 | 392.13 |
|  | 120d | 7 | 124.73 | 102.91 | 169.98 | 49.64 | 179.41 |
